# Factors influencing job loss and early retirement in working men with prostate cancer—findings from the population-based Life After Prostate Cancer Diagnosis (LAPCD) study

**DOI:** 10.1007/s11764-018-0704-x

**Published:** 2018-07-30

**Authors:** Damien Bennett, Therese Kearney, David W. Donnelly, Amy Downing, Penny Wright, Sarah Wilding, Richard Wagland, Eila Watson, Adam Glaser, Anna Gavin

**Affiliations:** 1 Northern Ireland Cancer Registry, Mulhouse Building, Queen’s University Belfast, Mulhouse Rd., Belfast, BT12 6DP Northern Ireland; 2 Leeds Institute of Cancer and Pathology, University of Leeds, Leeds, LS2 9JT UK; 3 Leeds Institute of Data Analytics, University of Leeds, Leeds, LS2 9JT UK; 4 Faculty of Health Sciences, University of Southampton, Southampton, UK; 5 Faculty Health and Life Sciences, Oxford Brookes University, Oxford, OX3 0BP UK

**Keywords:** Prostate cancer, Unemployment, Retirement, LAPCD

## Abstract

**Purpose:**

To investigate factors associated with job loss and early retirement in men diagnosed with prostate cancer (PCa) 18–42 months previously.

**Methods:**

Men ≤ 60 years at diagnosis who completed the Life After Prostate Cancer Diagnosis (LAPCD) survey were identified. Men who moved from employment at diagnosis to unemployment (EtoU) or retirement (EtoR) at survey (18–42 months post-diagnosis) were compared to men remaining in employment (EtoE). Sociodemographic, clinical and patient-reported factors were analysed in univariable and multivariable analysis.

**Results:**

There were 3218 men (81.4%) in the EtoE, 245 (6.2%) in EtoU and 450 (11.4%) in the EtoR groups. Men with stage IV disease (OR = 4.7 95% CI 3.1–7.0, relative to stage I/II) and reporting moderate/big bowel (OR = 2.5, 95% CI 1.6–3.9) or urinary problems (OR = 2.0, 95% CI 1.4–3.0) had greater odds of becoming unemployed. Other clinical (≥ 1 comorbidities, symptomatic at diagnosis) and sociodemographic (higher deprivation, divorced/separated), living in Scotland or Northern Ireland (NI)) factors were predictors of becoming unemployed. Men who were older, from NI, with stage IV disease and with caring responsibilities had greater odds of retiring early. Self-employed and non-white men had lesser odds of retiring early.

**Conclusion:**

PCa survivors who retire early following diagnosis do not report worse urinary or bowel problems compared to men remaining in employment. However, we identified clinical and sociodemographic factors which increased unemployment risk in PCa survivors.

**Implications for Cancer Survivors:**

Targeted support and engagement with PCa survivors at risk of unemployment, including their families and employers, is needed.

**Electronic supplementary material:**

The online version of this article (10.1007/s11764-018-0704-x) contains supplementary material, which is available to authorized users.

## Background

As the diagnosis, treatment and survival of most cancers have improved, the number of cancer survivors has increased, with this trend set to continue [1]. Prostate cancer (PCa) survivors account for a large proportion of these, with 30% of UK cancer survivors living with the disease [2]. Although PCa incidence is more common in older men, approximately 30% of UK survivors are of working age and the effect of cancer and its treatment can adversely impact working life and employment status [3, 4]. Indeed, a UK study found the greatest increase in PCa incidence rates between 2000 and 2010 was in men under 60 years of age [5].

Recently, there has been increased focus on workers diagnosed with cancer to ensure that appropriate assistance and information is given to support decisions about work and personal finances [6]. Work can be important for men recovering from cancer as it allows them support themselves and their families, socialise with colleagues and regain a sense of normality which can help them to ‘move on’ [7, 8]. Studies of risk factors for job loss in PCa survivors have involved small numbers of PCa survivors and usually been part of larger cancer cohorts from Nordic countries, the USA or Australia [9]. Although previous studies describe demographic, clinical and work-related characteristics associated with work ability, employment status and return to work for cancer survivors across a range of tumour sites, variable findings have been reported and few focus specifically on PCa survivors [9]. A study which reported on 100 PCa survivors in Ireland 6–24 months post-diagnosis found those who were self-employed had lower household income and did not have surgery were more likely to continue working following diagnosis, while those with lower educational level, medical card entitlement (providing free access to public health services) and not receiving sick pay were more likely not to resume work following diagnosis [10]. However, the effect of recent diagnosis of PCa on subsequent employment, and particularly unemployment and early retirement, have not been previously reported in a large-scale study. This study aimed to identify factors associated with movement from employment to unemployment or early retirement in working age men diagnosed with PCa in the UK.

## Methods

Data were collected as part of the UK-wide Life After Prostate Cancer Diagnosis (LAPCD) study. The study design has been reported elsewhere [11]. PCa survivors between 18 and 42 months following first diagnosis were identified from population-based cancer registries in England, Wales and Northern Ireland (NI) and from hospital activity data in Scotland. A postal questionnaire was sent to 58,930 men. Respondents answered questions on functional outcomes and personal and sociodemographic characteristics and other measures including health-related quality of life (HRQL), social difficulties, decision regret and emotional well-being.

Men aged 60 years and younger at time of diagnosis who completed a questionnaire were included in this study. The UK state pension age for men at the time of survey (October 2015 to November 2016) was 65 years [12] and the average age of men withdrawing from the labour market was just below 65 years (64.6 years) [13]. Consequently, we used a practical threshold of 60 years and below at diagnosis for inclusion. Although it is difficult to define early retirement, men aged 60 years and below at diagnosis would have been aged up to 63.5 years when they participated in LAPCD (18–42 months later), below the UK male state pension age. In the UK, currently only 22% of men aged 60 and below have retired suggesting this as a reasonable cutoff [13].

Responses to questions about employment status at time of cancer diagnosis and time of survey were used to categorise men as moving from employment to unemployment (EtoU), from employment to retirement (EtoR) or remaining in employment (EtoE) (Survey in Supplementary File 1). Those who chose ‘full time employment’ (FTE), ‘part time employment’ (PTE) or ‘self-employed’ (SE) were categorised as ‘employed’ while those who chose ‘unemployed, seeking work’ or ‘unemployed, unable to work for health reasons’ were categorised as ‘unemployed’ and those who chose ‘retired’ were classified as such. Those who recorded ‘looking after family/home’ and ‘other’ were excluded from analysis as focus was on movement between employed and unemployed and retired states and it would be difficult to clearly delineate a change in status between these states and the unemployed or retired state.

### Clinical characteristics, sociodemographic factors and patient-reported symptoms

Stage and age at diagnosis and UK nation of residence were determined from cancer registration data. Deprivation levels were determined from UK Indices of Multiple Deprivation (IMD) derived from patients’ home postcode at diagnosis [14–17]. Respondents’ self-reported employment status; relationship status; ethnicity; height and weight (from which BMI was derived [18]); whether they had carer responsibilities; whether they had ever seen a healthcare professional for problems with emotions; nerves or use of alcohol or drugs; treatment type; comorbidities (total number of long-term conditions (LTCs), e.g. stroke, diabetes) and overall urinary and bowel function (from the Expanded Prostate Cancer Index Composite short form (EPIC-26) questions ‘How big a problem has your urinary/bowel function been for you during the last 4 weeks?’ [19] were taken from the survey data (Supplementary File 1).

### Statistical analysis

Univariable analyses were undertaken to assess differences in sociodemographic and clinical characteristics between both the EtoU and EtoR groups and the EtoE group. Differences in categorical variables were assessed using Chi-squared tests and continuous variables using *t* tests. Bonferroni correction was used to compensate for multiple comparisons. Variables were entered as predictors in regression analysis using a univariable analysis cutoff of *p* < 0.2 or if they were of a priori importance (e.g., age, patient-reported symptoms). Treatment type (surgery, radiotherapy, etc.) was not included in regression analysis. Certain treatments are more likely to lead to specific function problems (e.g., surgery is associated with worse urinary function) and it is the effect of resulting symptoms that is of interest. Multivariable logistic regression (backwards stepwise) analyses was performed with outcome variables being change in employment status from EtoU and from EtoR with the reference category being no change in employment status (i.e. EtoE). Data were analysed with SPSS Version 22.0 (IBM Corp, Armonk, NY).

## Results

Of the 58,930 men invited to participate, 35,823 returned completed questionnaires (60.8% response rate);14.1% (*N* = 5037/35,823) of respondents were ≤ 60 years at PCa diagnosis. Non-response on employment status was low: 1.5% (75/5037) did not respond on employment status at diagnosis, 2.2% (*N* = 109) did not respond on employment status at time of survey and 3.1% (*N* = 155) did not respond on both. Non-responders were more likely to be divorced, living in areas of greater deprivation, of non-white ethnicity and report overall urinary problems. Supplementary Table 1 details the characteristics of employment status respondents and non-respondents.

**Fig. 1:**
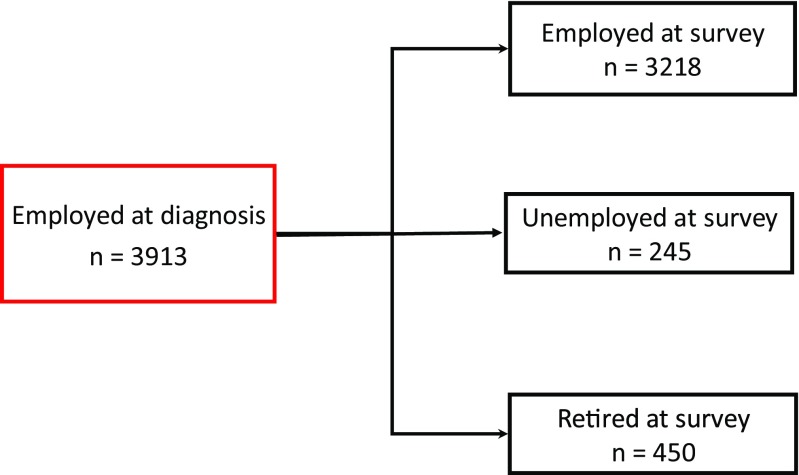
Schematic of men who were employed at diagnosis and employed, unemployed or retired at time of survey

Employment status and change in employment status between time of diagnosis and time of survey are shown in Table 1. Of those aged ≤ 60 years at diagnosis, 4014 were employed at diagnosis and 3913 of these were employed, unemployed or retired at survey. Of these 3913 men, there were 3218 (81.4%) in the EtoE group, 245 (6.2%) in the EtoU group and 450 (11.4%) in the EtoR group (Fig. 1). Table 2 details the characteristics, treatment and patient-reported symptoms variables in the three groups.

**Table 1:** Employment status at time of diagnosis and survey, and change in status, for men aged 60 years old and less at time of diagnosis

	Employed	Retired	Unemployed	Home	Other	Total	Missing
At diagnosis	80.9%	10.4%	7.1%	0.8%	0.8%	100%	
	(4014)	(516)	(351)	(42)	(39)	(4962)	75
At survey	66.7%	19.8%	11.3%	0.8%	1.3%	100%	
	(3289)	(977)	(559)	(40)	(63)	(4928)	109
	Employed to employed (EtoE)	Employed to retired (EtoR)	Employed to unemployed (EtoU)	Employed to home (EtoH)	Employed to other (EtoO)	Total	Missing
Change in employment status between diagnosis and survey							
% (number)	81.4%	11.4%	6.2%	0.3%	0.8%	100%	
	(3218)	(450)	(245)	(10)	(32)	(3955)	59

**Table 2:** Sociodemographic, clinician and patient-reported urinary and bowel symptoms for EtoE, EtoU and EtoR groups for men aged 60 years and below

Variable	Employed to employed (EtoE)	Employed to unemployed (EtoU)	*p* value (comparing EtoE and EtoU)	Total	Employed to retired (EtoR)	*p* value (comparing EtoE and EtoR)	Total
Mean age (years) [SD]	55.4 [3.8]	55.9 [3.5]	0.03 (*t* test)		57.7 [2.5]	< 0.001* (*t* test)	
Age bands			0.290			< 0.001*	
≤ 50 years	11.0% (353)	8.2% (20)		10.8% (373)	1.6% (7)		9.8% (360)
51–55	33.2% (1067)	31.8% (78)		33.1% (1145)	15.3% (69)		31.0% (1136)
56–60	55.9% (1798)	60.0% (147)		56.2% (1945)	83.1% (374)		59.2% (2172)
Total	100% (3218)	100% (245)		100% (3463)	100% (450)		100% (3668)
Marital status			< 0.001*			0.036	
Married/civil partnership	80.9% (2598)	68.3% (166)		80.0% (2764)	81.1% (365)		80.9% (2963)
Divorced/separated	10.1% (324)	19.8% (48)		10.8% (372)	8.0% (36)		9.8% (360)
Widowed	1.5% (47)	1.6% (4)		1.5% (51)	2.7% (12)		1.6% (59)
Single	4.7% (152)	7.0% (17)		4.9% (169)	6.7% (30)		5.0% (182)
Other	2.8% (90)	3.3% (8)		2.8% (98)	1.6% (7)		2.6% (97)
Total	100% (3211)	100% (243)		100% (3454)	100% (450)		100% (3661)
Deprivation			< 0.001*			0.009	
Q1 (Area of least deprivation)	27.9% (876)	16.3% (39)		27.0% (915)	33.9% (151)		28.6% (1027)
Q2	26.2% (825)	19.2% (46)		25.7% (871)	26.5% (118)		26.3% (943)
Q3	18.7% (587)	18.8% (45)		18.7% (632)	19.1% (85)		18.7% (672)
Q4	16.0 (503)	21.3% (51)		16.4% (554)	13.9% (62)		15.7% (565)
Q5 (area of greatest deprivation)	11.3% (354)	24.6% (59)		12.2% (413)	6.7% (30)		10.7% (384)
Total	100% (3145)	100% (240)		100% (3385)	100% (446)		100% (3591)
UK country of residence			< 0.001*			0.003	
England	85.3% (2744)	75.9% (186)		84.6% (2930)	81.6% (367)		84.8% (3111)
Wales	7.0% (225)	6.5% (16)		7.0% (241)	8.9% (40)		7.2% (265)
Scotland	4.5% (146)	9.8% (24)		4.9% (170)	3.3% (15)		4.4% (161)
Northern Ireland	3.2% (103)	7.8% (19)		3.5% (122)	6.2% (28)		3.6% (131)
Total	100% (3218)	100% (245)		100% (3463)	100% (450)		100% (3668)
Ethnicity			0.794			< 0.001*	
White	92.6% (2938)	93.3% (223)		92.7% (3161)	97.5% (434)		93.2% (3372)
Non-white	7.4% (234)	6.7% (16)		7.3% (250)	2.5% (11)		6.8% (245)
Total	100% (3172)	100% (239)		100% (3411)	100% (445)		100% (3617)
Stage at diagnosis			< 0.001*			0.053	
I/II	72.2% (2020)	50.5% (105)		70.7% (2125)	69.5% (267)		72.2% (2020)
III	20.9% (584)	23.6% (49)		21.1% (633)	20.1% (77)		20.8% (661)
IV	7.0% (195)	26.0% (54)		8.3% (249)	10.4% (40)		7.4% (235)
Total	100% (2799)	100% (208)		100% (3007)	100% (384)		100% (3183)
Treatment type			< 0.001*			0.071	
Active surveillance and watchful waiting	16.1% (517)	4.5% (11)		15.3% (528)	15.8% (71)		16.0% (588)
Surgery	41.5% (1336)	25.7% (63)		40.4% (1399)	35.3% (159)		40.8% (1495)
ERBT	2.5% (81)	4.1% (10)		2.6% (91)	2.2% (10)		2.5% (91)
Brachytherapy	6.9% (221)	4.9% (12)		6.7% (233)	7.6% (34)		7.0% (255)
ADT	1.3% (42)	3.7% (9)		1.5% (51)	2.9% (13)		1.5% (55)
EBRT + ADT	9.9% (318)	16.3% (40)		10.3% (358)	12.4% (56)		10.2% (374)
Surgery + EBRT/ADT	8.2% (265)	9.4% (23)		8.3% (288)	8.9% (40)		8.3% (305)
ADT + systemic treatment	1.0% (32)	4.1% (10)		1.2% (42)	1.8% (8)		1.1% (40)
EBRT + systemic treatment	1.4% (45)	6.1% (15)		1.7% (60)	1.6% (7)		1.4% (52)
Other	11.2% (360)	21.2% (52)		11.9% (412)	11.6% (52)		11.2% (412)
Total	100% (3217)	100% (245)		100% (3462)	100% (450)		100% (3667)
Comorbidities			< 0.001*			0.003*	
None	47.7% (1536)	27.3% (67)		46.3% (1603)	40.4% (182)		46.8% (1718)
1	33.3% (1070)	33.9% (83)		33.3% (1153)	36.4% (164)		33.6% (1234)
2	12.1% (389)	20.4% (50)		12.7% (439)	12.2% (55)		12.1% (444)
3	3.6% (115)	9.0% (22)		4.0% (137)	6.7% (30)		4.0% (145)
4 or more	3.4% (108)	9.4% (23)		3.8% (131)	4.2% (19)		3.5% (127)
Total	100% (3218)	100% (245)		100% (3463)	100% (450)		100% (3668)
Symptomatic at diagnosis			< 0.001*			0.713	
No	48.9% (1555)	31.8% (76)		47.7% (1631)	47.9% (214)		48.8% (1769)
Yes	51.1% (1623)	68.2% (163)		52.3% (1786)	52.1% (233)		51.2% (1856)
Total	100% (3178)	100% (239)		100% (3417)	100% (447)		100% (3625)
Overall urinary symptoms			< 0.001*			0.831	
No/very small/small problem	89.4% (2868)	69.0% (167)		88.0% (3035)	89.9% (400)		89.5% (3268)
Moderate/big problem	10.6% (339)	31.0% (75)		12.0% (414)	10.1% (45)		10.5% (384)
Total	100% (3207)	100% (242)		100% (3449)	100% (445)		100% (3652)
Overall bowel symptoms			< 0.001*			0.614	
No/very small/small problem	94.7% (3034)	77.3% (187)		93.4% (3221)	94.0% (420)		94.6% (3454)
Moderate/big problem	5.3% (171)	22.7% (55)		6.6% (226)	6.0% (27)		5.4% (198)
Total	100% (3205)	100% (242)		100% (3447)	100% (447)		100% (3652)
BMI			< 0.001*			0.092	
< 25 kg/m^2^	26.7% (810)	21% (47)		26.3% (857)	30% (131)		27.1% (941)
25–29.9 kg/m^2^	48% (1459)	40.2% (90)		47.5% (1549)	49.1% (214)		48.1% (1673)
≥ 30 kg/m^2^	25.3% (770)	38.8% (87)		26.3% (857)	20.9% (91)		24.8% (861)
Total	100% (3039)	100% (224)		100% (3263)	100% (436)		100% (3475)
Employment type			0.029			< 0.001*	
Full time	72.3% (2327)	73.1% (179)		72.4% (2506)	77.8% (350)		73% (2677)
Part time	5.4% (174)	9% (22)		5.7% (196)	10.7% (48)		6.1% (222)
Self-employed	22.3% (717)	18% (44)		22% (761)	11.6% (52)		21% (769)
Total	100% (3218)	100% (245)		100% (3463)	100% (450)		100% (3668)
Seen HC professional for mental health issues^‡^			< 0.001*			0.183	
Yes	22% (700)	33.7% (82)		22.8% (782)	24.8% (111)		22.3% (811)
No	78% (2489)	66.3% (161)		77.2% (2650)	75.2% (337)		77.7% (2826)
Total	100% (3189)	100% (243)		100% (3432)	100% (448)		100% (3637)
Caring responsibilities			0.875			0.003*	
Yes	22.3% (707)	22.9% (55)		22.3% (762)	28.7% (127)		23% (834)
No	77.7% (2470)	77.1% (185)		77.7% (2655)	71.3% (316)		77% (2786)
Total	100% (3177)	100% (240)		100% (3417)	100% (443)		100% (3620)

*EBRT* external beam radiotherapy, *ADT* androgen deprivation therapy

*Significant at *p* < 0.05 after Bonferroni adjustment for multiple comparisons

^‡^Ever seen a healthcare professional for problems with emotions or nerves or use of alcohol or drugs

## Men becoming unemployed

There was no difference in the proportion of EtoU men aged 56–60 years (60.0%) compared to EtoE men (55.9%) (*p* = 0.29) (Table 2). Univariable analysis demonstrated there were greater proportions of men who became unemployed who were divorced, from deprived areas, from Scotland or NI, with late stage disease at diagnosis, symptomatic at diagnosis, with more comorbidities and reporting moderate or big problems with urinary and bowel function (Table 2). There was no difference in ethnicity between EtoE and EtoU groups (Table 2). A lower proportion of EtoU men had surgery and experienced active surveillance, but a greater proportion had external beam radiotherapy (EBRT) and androgen deprivation therapy (ADT).

Multivariable logistic regression demonstrated a range of sociodemographic, clinical and patient-reported factors were predictive of movement from employment at diagnosis to unemployment at follow-up (i.e. comparing EtoU to EtoE groups) (Table 3). Late stage at diagnosis (OR = 4.7 (95% CI 3.1–7.0), stage IV relative to stage I/II) and greater comorbidity (OR ranging from OR 1.6 (95% CI 1.1–2.3) for 1 LTC to 3.5 (95% CI 1.8–6.8) for ≥ 4 LTCs compared to none) were the strongest predictors of movement to unemployment. Problems with bowel (OR = 2.5 (95% CI 1.6–3.9) moderate/big compared to no/very small/small problems) and urinary function (OR = 2.0 (95% CI 1.4–2.9) moderate/big compared to no/very small/small problems) and having symptoms at diagnosis (OR = 1.5 (95% CI 1.0–2.1)) were also predictors of movement to unemployment (Table 3). Living in areas of greater deprivation (OR = 2.6 (95% CI 1.6–4.3] most relative to least deprived), being divorced/separated (OR = 2.5 (95% CI 1.7–3.8]) and living in Scotland (OR = 2.1 (95% CI 1.2–3.6]) or NI (OR = 3.1 (95% CI 1.7–5.6] compared to living in England) were also significant predictors of becoming unemployed.

**Table 3:** Significant independent predictors of movement between employment and unemployment for men aged 60 years and below using logistic regression modelling

	Odds ratio	95% CI lower	95% CI upper	*p* value
Deprivation				
Q1 (area of least deprivation)	1.00			
Q2	0.87	0.50	1.50	0.618
Q3	1.57	0.93	2.63	0.090
Q4	1.92	1.16	3.19	0.011*
Q5 (area of greatest deprivation)	2.58	1.56	4.26	< 0.001*
Marital status				
Married/civil partnership	1.00			
Divorced/separated	2.50	1.65	3.80	< 0.001*
Widowed	1.58	0.51	4.93	0.42
Single	1.51	0.80	2.87	0.205
Other	1.29	0.52	3.16	0.580
UK country				
England	1.00			
Wales	1.08	0.57	2.05	0.811
Scotland	2.08	1.20	3.61	0.009*
Northern Ireland	3.11	1.71	5.64	< 0.001*
Stage				
Stage I/II	1.00			
Stage III	1.72	1.17	2.53	0.006*
Stage IV	4.68	3.11	7.03	< 0.001*
Symptomatic at diagnosis				
No	1.00			
Yes	1.47	1.04	2.06	0.028*
Comorbidities				
No comorbidities	1.00			
1 comorbidity	1.57	1.07	2.32	0.023
2 comorbidities	2.27	1.44	3.57	< 0.001*
3 comorbidities	2.47	1.30	4.68	0.006*
4 or more comorbidities	3.49	1.80	6.79	< 0.001*
Bowel symptoms (overall)				
No/very small/small problems	1.00			
Moderate/big problems	2.54	1.64	3.94	< 0.001*
Urinary symptoms (overall)				
No/very small/small problems	1.00			
Moderate/big problems	2.02	1.37	2.97	< 0.001*

Factors contributing significantly to the model (p < 0.05) are reported. Variables included in the model were age, relationship status, deprivation quintile, UK country of residence, ethnicity, BMI, type of employment at diagnosis, stage at diagnosis, whether symptomatic at diagnosis, whether had PSA testing at diagnosis, comorbidities, overall urinary problems, overall bowel problems, having ever seen a professional for mental health issues and caring responsibilities

## Men retiring early

In the univariable analysis, there were no differences in disease stage at diagnosis or the proportions who were symptomatic at diagnosis between men retiring early and those who remained in employment (Table 2). There were no differences in treatment type or overall urinary or bowel function between the EtoR and EtoE group. Men remaining in employment were significantly younger (mean age = 55.4 years, *p* < 0.001) than those retiring early (mean age = 57.7 years), with a difference of over 2 years between the groups. There was a greater proportion of older men in the EtoR group with 83.1% aged 56–60 years compared to 55.9% in the EtoE group (Table 2). There were greater proportions of EtoR relative to EtoE men of white ethnicity, from less-deprived areas and with caring responsibility and lower proportions of EtoR compared to EtoE men living in England and reporting no comorbidities (Table 2).

In the multivariable analysis, age, ethnicity, employment status, UK country of residence, stage at diagnosis and carer responsibilities were significantly associated with moving from employment to retirement (Table 4). Older age was the strongest predictor of early retirement (OR 8.5 (95% CI 4.0–18.3), age 55–60 years). Men living in NI (OR = 2.3 (95% CI 1.4–3.6)), with later disease stage disease (OR = 1.8 (95% CI 1.2–2.6), stage IV) and carer responsibilities (OR = 1.3 (95% CI 1.0–1.7)) were also significantly more likely to move to early retirement. Men who were self-employed (OR = 0.40 (95% CI 0.28–0.57)) and of non-white ethnicity (OR = 0.32 (95% CI 0.16–0.63)) were significantly less likely to move to early retirement.

**Table 4:** Significant independent predictors of movement between employment and retirement for men aged 60 years and below using logistic regression modelling

	Odds ratio	95% CI lower	95% CI upper	*p* value
Age band				
< 50 years	1.00			
50–54 years	2.57	1.16	5.71	0.021
55–60 years	8.52	3.97	18.28	< 0.001*
Ethnicity (reference)				
White	1.00			
Non-white	0.32	0.16	0.63	0.001*
Employment type				
Full-time employment	1.00			
Part-time employment	1.38	0.94	2.03	0.099
Self-employed	0.40	0.28	0.57	< 0.001*
UK country				
England	1.00			
Wales	1.25	0.83	1.87	0.281
Scotland	0.67	0.36	1.25	0.208
Northern Ireland	2.29	1.44	3.64	< 0.001*
Stage				
Stage I/ II	1.00			
Stage III	0.99	0.75	1.31	0.934
Stage IV	1.79	1.22	2.62	0.003*
Carer responsibilities				
No	1.00			
Yes	1.29	1.00	1.66	0.050*

Factors contributing significantly to the model (*p* < 0.05) are reported. Variables included in the model were age, relationship status, deprivation quintile, UK country of residence, ethnicity, BMI, type of employment at diagnosis, stage at diagnosis, comorbidities, overall urinary problems, overall bowel problems, having ever seen a professional for mental health issues and caring responsibilities

## Discussion

The clinical factors of advanced disease stage, presence of bowel and urinary problems, having symptoms at diagnosis and greater levels of comorbidity increased the odds of job loss in PCa survivors, alongside the sociodemographic factors of deprivation, divorce/separation and living in Scotland or NI. In contrast, having bowel or urinary problems or greater comorbidity were not significantly associated with early retirement in PCa survivors. Men who were older, of white ethnicity, in full-time employment, with most advanced disease (stage IV) or with caring responsibilities had greater odds of early retirement.

### Movement to unemployment

In our study, advanced disease stage at diagnosis was the strongest predictor of becoming unemployed, with the odds of men with stage IV disease becoming unemployed almost five times those of men with stage I/II disease. Problems with bowel and urinary function were also strong predictors of becoming unemployed. This suggests that more severe disease and treatment side effects adversely impact on employment. Previous studies of cancer survivors report associations between both cancer severity and adverse effects and delayed returning to work [20]. However, those involving PCa survivors report variable associations between clinical factors and employment status, productivity and work engagement [21–24]. However, these studies were not population based, involved small numbers of PCa survivors (*n* < 180) and were non-UK based. More severe bowel symptoms have, for example, been associated with greater number of missed workdays in irritable bowel syndrome (IBS) sufferers [25], who have been reported to experience significant work impairment with substantial productivity and cost implications [26, 27]. Comorbidity was also a significant predictor of becoming unemployed with the likelihood of job loss increasing with the number of comorbidities. Greater comorbidity has been associated with reduced work ability in Nordic PCa survivors [28], but greater disability was not associated with higher job quitting rates in a US study of PCa survivors [29].

We have identified sociodemographic factors that predict job loss. Men living in the most deprived areas had over twice the odds of becoming unemployed. A study reporting on 100 PCa survivors in Ireland found more socioeconomically deprived men were less likely to resume work following diagnosis [10]. A possible reason may have been difficulty maintaining physically demanding or manual jobs, which are more common in men in deprived areas [30]. Cancer survivors with physically demanding jobs, such as heavy lifting, found their jobs more challenging [31, 32], and manual labour was found to negatively impact on survivors return-to-work [33]. As men from more deprived areas may have more physically demanding or manual jobs this may have been a possible reason for their higher odds of unemployment. Divorced or separated men had 2.5 times greater odds of becoming unemployed compared to married men. Men living in NI and Scotland were over twice as likely to become unemployed as English men, which broadly reflect unemployment patterns between 2013 and 2016 in which, against a background downward trend in all UK countries, unemployment rates in NI and Scotland were slightly higher than the UK average [34].

### Early retirement

Our findings for men who moved from employment to early retirement were very different, and men with more severe urinary and bowel problems or a greater number of comorbidities were not more likely to retire early. Older age was the strongest predictor of early retirement with the odds of retiring early for men aged 55–60 years almost nine times greater than that of men less than 50 years old, although numbers in the reference category (< 50 years) were small (*N* = 20). It is not surprising that older men were more likely to retire than younger men in this study. However, older men were not more likely to be become unemployed, with no significant relationship between age and movement to unemployment on logistic regression analysis.

Those of non-white ethnicity were significantly less likely to retire early, although numbers in this group were small. Male ethnic minorities in the UK experience higher rates of unemployment [35] and have, on average, lower income than the white population [36], with earning differentials at least 10% less than comparable white men [37]. Consequently, white UK men may be more economically able to support themselves and their families in retirement at an earlier age. Employment type (e.g. full-time, part-time, self-employed) predicted move to early retirement, but not to unemployment. The odds of self-employed men moving to early retirement were 60% less than men in full-time employment, which supports the finding of Sharp et al. that self-employed PCa survivors were more likely to continue working following diagnosis [10].

The findings from the present study suggest that men who became unemployed and those who retired early following PCa diagnosis represent very different groups. Compared with men who remain in employment, those who become unemployed had different socioeconomic characteristics, more severe disease and worse clinical symptoms. In contrast, the sociodemographic profile and symptom characteristics of men who retire early was similar to men who continue to work. Men with PCa who retired early had no worse urinary or bowel symptoms than those who remain in employment. They were less likely to be self-employed and not more deprived. However, they are more likely to have caring responsibilities, suggesting this may be a possible reason for retiring early. Although it is unsurprising that those from more deprived areas had greater odds of becoming unemployed and those from less-deprived areas had greater odds of retiring early, there are few studies which consider factors associated with change in employment status of cancer survivors following diagnosis (i.e. moving from employment to either unemployment or early retirement compared to those who stay in employment) and none involving a population-based study of prostate cancer survivors.

### Practical implications

This study has identified characteristics associated with job loss and early retirement, which can be used by health and social care staff, employers and human resources staff to target information, advice and support for PCa survivors to mitigate work impairment and support return to work. Local networks integrated with existing care systems have been suggested as a model, which can support urological cancer survivors who wish to stay in employment [38] and workplace counselling [39], workplace evaluation [40] and workplace rehabilitation [41] may help sustain cancer survivors in employment. Ideally, PCa survivors at risk of unemployment would be empowered to actively seek assistance, which could maintain employment.

Factors associated with employment outcomes for cancer survivors include health and well-being, symptom control and function as well as work demands, work environment and policies and economic factors [42]. Our study found that men with worse urinary and bowel symptoms had greater odds of becoming unemployed. Men with a greater number of comorbidities, and likely poorer function, also had greater odds of losing their job. Men who were divorced/separated or living in deprived areas also had greater odds of losing their job. Targeted support to maintain employment may be warranted for PCa survivors who are, for example, divorced or separated, from more deprived backgrounds and with a number of comorbidities, especially those experiencing urinary or bowel symptoms following treatment. This study will also help inform future research into maintaining employment following PCa diagnosis. Such research could focus on disease-specific symptoms and general health status alongside social and demographic factors, ideally in longitudinal studies which would also consider work-related factors.

### Limitations

Although this is the first study to investigate a range of sociodemographic, clinical and patient-reported factors associated with movement from employment at time of PCa diagnosis to unemployment or early retirement in a large-scale UK population-based study, there were a number of limitations. We did not have details of when men became unemployed, of income, pension or educational levels or of work-related factors, such as type of occupation (e.g. service and manufacturing) and hours worked, which may have been associated with movement to unemployment or early retirement. Although we report significant associations with movement to unemployment or early retirement, we cannot assume causal relationships. We acknowledge that some of the observed movement to early retirement may have occurred independent of PCa diagnosis. We compared both EtoU and EtoR groups with recently diagnosed PCa survivors who were employed both at diagnosis and follow-up (EtoE group) and assumed these men remained in employment during that period. Some variables had low numbers in sub-categories (e.g. those of non-white ethnicity who retired early, *N* = 11) which warrant caution in interpretation. However, these were in the context of large category numbers (e.g. 261 men of non-white ethnicity in the sample) and overall group numbers (3218 in EtoE, 245 in EtoU and 450 in the EtoR groups) which were much greater than previous studies of job loss and early retirement involving PCa survivors.

## Conclusion

Men who retire early following PCa diagnosis do not report worse overall urinary or bowel problems or different socioeconomic characteristics to men remaining in employment. However, this study has identified risk factors for job loss in PCa survivors, which can be used to support men following diagnosis. Targeted support and engagement with these men, their families and their employers is needed.

## Electronic supplementary material

ESM 1 (DOCX 28 kb)

ESM 2 (DOCX 82 kb)
