# Supplementary material for: Factors influencing job loss and early retirement in working men with prostate cancer—findings from the population-based Life After Prostate Cancer Diagnosis (LAPCD) study
Source: J Cancer Surviv. 2018 Jul 30;12(5):669–78. doi: 10.1007/s11764-018-0704-x (PMC6153559; doi:10.1007/s11764-018-0704-x)
Supplement: Supplementary file 1 — (DOCX 28 kb) [file 11764_2018_704_MOESM1_ESM.docx]

**Supplementary Table 1. Characteristics of employment status respondents and non-respondents in cohort.**

| **Variable** | **Respondents** | **Non-Respondents** | **p value** | **Total** |
| --- | --- | --- | --- | --- |
| **Age bands** |  |  | 0.128 |  |
| ≤ 50 yrs | 8.9% (435) | 5.8% (9) |  | 8.8% (444) |
| 51-55 | 27.7% (1354) | 23.2% (36) |  | 27.6% (1390) |
| 56-60 | 63.4% (3093) | 71% (110) |  | 63.6% (3203) |
| Total | 100% (4882) | 100% (155) |  | 100% (5037) |
|  |  |  |  |  |
| **Marital status** |  |  | 0.025 |  |
| Married/civil partnership | 77.9% (3790) | 68.2% (90) |  | 77.6% (3880) |
| Divorced/separated | 11% (534) | 17.4% (23) |  | 11.1% (557) |
| Widowed | 1.8% (86) | 1.5% (2) |  | 1.8% (88) |
| Single | 6.6% (320) | 6.8% (9) |  | 6.6% (329) |
| Other | 2.8% (136) | 6.1% (8) |  | 2.9% (144) |
| Total | 100% (4866) | 100% (132) |  | 100% (4998) |
|  |  |  |  |  |
| **Deprivation** |  |  | 0.004 |  |
| Q1 (Least Deprived) | 26.7% (1272) | 22.5% (34) |  | 26.5% (1306) |
| Q2 | 24.6% (1173) | 19.9% (30) |  | 24.4% (1203) |
| Q3 | 19.2% (914) | 17.9% (27) |  | 19.1% (941) |
| Q4 | 16.6% (792) | 15.9% (24) |  | 16.6% (816) |
| Q5 (Most Deprived) | 13% (620) | 23.8% (36) |  | 13.3% (656) |
| Total | 100% (4771) | 100% (151) |  | 100% (4922) |
|  |  |  |  |  |
| **UK country of residence** |  |  | 0.402 |  |
| England | 83.7% (4086) | 84.5% (131) |  | 83.7% (4217) |
| Wales | 7.4% (362) | 9.7% (15) |  | 7.5% (377) |
| Scotland | 4.9% (239) | 2.6% (4) |  | 4.8% (243) |
| Northern Ireland | 4% (195) | 3.2% (5) |  | 4% (200) |
| Total | 100% (4882) | 100% (155) |  | 100% (5037) |
|  |  |  |  |  |
|  |  |  |  |  |
| **Ethnicity** |  |  | 0.008 |  |
| White | 93.5% (4500) | 87% (94) |  | 93.3% (4594) |
| Ethnic minority (Black African, Asian, Gypsy, Arab etc.) | 6.5% (314) | 13% (14) |  | 6.7% (328) |
| Total | 100% (4814) | 100% (108) |  | 100% (4922) |
|  |  |  |  |  |
| **Stage at diagnosis** |  |  | 0.890 |  |
| I / II | 70.5% (2984) | 71.7% (99) |  | 70.5% (3083) |
| III | 20.5% (868) | 18.8% (26) |  | 20.4% (894) |
| IV | 9% (382) | 9.4% (13) |  | 9% (395) |
| Total | 100% (4234) | 100% (138) |  | 100% (4372) |
|  |  |  |  |  |
| **Treatment type †** |  |  | 0.167 |  |
| Active Surveillance and Watchful waiting | 15.3% (746) | 11.6% (18) |  | 15.2% (764) |
| Surgery | 38.2% (1865) | 31.6% (49) |  | 38% (1914) |
| ERBT | 3% (145) | 4.5% (7) |  | 3% (152) |
| Brachytherapy | 6.3% (306) | 4.5% (7) |  | 6.2% (313) |
| ADT | 1.7% (85) | 3.9% (6) |  | 1.8% (91) |
| EBRT + ADT | 12.1% (593) | 12.9% (20) |  | 12.2% (613) |
| Surgery + EBRT/ADT | 8% (391) | 10.3% (16) |  | 8.1% (407) |
| ADT + Systemic Therapy | 1.3% (64) | 1.3% (2) |  | 1.3% (66) |
| EBRT + Systemic Therapy | 1.6% (79) | 2.6% (4) |  | 1.6% (83) |
| Other | 12.4% (607) | 16.8% (26) |  | 12.6% (633) |
| Total | 100% (4881) | 100% (155) |  | 100% (5036) |
|  |  |  |  |  |
| **Co morbidities** |  |  | 0.120 |  |
| None | 42.7% (2085) | 50.3% (78) |  | 42.9% (2163) |
| 1 | 33.7% (1645) | 23.9% (37) |  | 33.4% (1682) |
| 2 | 13.7% (668) | 16.1% (25) |  | 13.8% (693) |
| 3 | 5.3% (260) | 4.5% (7) |  | 5.3% (267) |
| 4 or more | 4.6% (224) | 5.2% (8) |  | 4.6% (232) |
| Total | 100% (4882) | 100% (155) |  | 100% (5037) |
|  |  |  |  |  |
| **Symptomatic at diagnosis** |  |  | 0.683 |  |
| No | 46.7% (2253) | 48.4% (74) |  | 46.7% (2327) |
| Yes | 53.3% (2572) | 51.6% (79) |  | 53.3% (2651) |
| Total | 100% (4825) | 100% (153) |  | 100% (4978) |
|  |  |  |  |  |
| **PSA testing at diagnosis** |  |  | 0.341 |  |
| No | 62% (2990) | 58.2% (89) |  | 61.9% (3079) |
| Yes | 38% (1835) | 41.8% (64) |  | 38.1% (1899) |
| Total | 100% (4825) | 100% (153) |  | 100% (4978) |
|  |  |  |  |  |
| **Overall Urinary symptoms** |  |  | <0.001 |  |
| No / very small / small problem | 86.5% (4205) | 76.3% (116) |  | 86.2% (4321) |
| Moderate / big problem | 13.5% (654) | 23.7% (36) |  | 13.8% (690) |
| Total | 100% (4859) | 100% (152) |  | 100% (5011) |
|  |  |  |  |  |
| **Overall Bowel symptoms** |  |  | 0.054 |  |
| No / very small / small problem | 92.2% (4477) | 87.9% (131) |  | 92.1% (4608) |
| Moderate / big problem | 7.8% (377) | 12.1% (18) |  | 7.9% (395) |
| Total | 100% (4854) | 100% (149) |  | 100% (5003) |
|  |  |  |  |  |
| **BMI** |  |  | 0.571 |  |
| < 25 kg/m^2^ | 26.9% (1240) | 22.5% (23) |  | 26.8% (1263) |
| 25-29.9 kg/m^2^ | 46.8% (2152) | 48% (49) |  | 46.8% (2201) |
| ≥ 30 kg/m^2^ | 26.3% (1210) | 29.4% (30) |  | 26.4% (1240) |
| Total | 100% (4602) | 100% (102) |  | 100% (4704) |
|  |  |  |  |  |
| **Seen HC professional for mental health issues ‡** |  |  | 0.946 |  |
| Yes | 26.1% (1263) | 26.4% (29) |  | 26.1% (1292) |
| No | 73.9% (3580) | 73.6% (81) |  | 73.9% (3661) |
| Total | 100% (4843) | 100% (110) |  | 100% (4953) |
|  |  |  |  |  |
| **Caring Responsibilities** |  |  | 0.932 |  |
| Yes | 24.7% (1187) | 24.3% (26) |  | 24.6% (1213) |
| No | 75.3% (3627) | 75.7% (81) |  | 75.4% (3708) |
| Total | 100% (4814) | 100% (107) |  | 100% (4921) |

Notes:

**†** EBRT = External Beam Radiotherapy. ADT = Androgen Deprivation Therapy.

**‡** Ever seen a healthcare professional for problems with emotions or nerves or use of alcohol or drugs.
