# Supplementary material for: Factors influencing job loss and early retirement in working men with prostate cancer—findings from the population-based Life After Prostate Cancer Diagnosis (LAPCD) study
Source: J Cancer Surviv. 2018 Jul 30;12(5):669–78. doi: 10.1007/s11764-018-0704-x (PMC6153559; doi:10.1007/s11764-018-0704-x)
Supplement: Supplementary file 2 — (DOCX 82 kb) [file 11764_2018_704_MOESM2_ESM.docx]

**Supplementary File 1 - Relevant Questions from LAPCD Survey**

**Prostate Patient Reported Outcomes Survey**

More men are now living longer after a diagnosis of prostate cancer. We want to find out what life is really like for this group of men. Your answers will benefit other men with prostate cancer in the future by providing information to help clinical teams, service providers and policy makers make decisions about how to improve the quality of services for prostate cancer patients. We would be grateful if you would complete this survey, which asks for information about your health and quality of life.

If you have **not** had a diagnosis of prostate cancer this questionnaire is not relevant to you. Please tick the “no” box below and please accept our apologies for contacting you. Please return the blank questionnaire in the envelope provided and we will correct our records. If you have any questions about this survey please contact the FREEPHONE helpline number: **0808 801 0678**.

**Have you ever been told by a doctor that you have prostate cancer?**

**Yes No**

If you have ticked yes to the first question please complete the rest of the survey.

If you have ticked no, please accept our apologies for this mistake, and send the questionnaire back to us in the envelope provided.

**The survey**

This survey is made up of eight sections and will take approximately 30 minutes to complete.

**Who should complete the questionnaire?**

The questions should be answered by the person named in the letter that came with this questionnaire. If that person needs help to answer the questions then the answers should be given from their point of view – not from the point of view of the person who is helping.

**Completing the questionnaire**

For each question please tick clearly inside the box of the response that best represents your views, using a black or blue pen. Do not worry if you make a mistake. Just cross out the mistake and put a tick in the correct box. Do not write your name or address anywhere on the questionnaire. The more questions in this survey that you complete, the more we can understand what life is like for those living with and beyond prostate cancer. However, if you feel unable or uncomfortable about answering any of the questions, leave it blank and move on to the next one.

The information you give us will be kept **securely** and treated in **confidence**. We will not publish any personal information that could allow anyone to identify you. We are very grateful for your time and effort in completing this survey.

If you have any queries about the questionnaire, please call the FREEPHONE helpline number: **0808 801 0678**

You can find more information about the study at <http://www.lifeafterprostatecancerdiagnosis.com/>

Section Two Your diagnosis and treatment

**7.** How were you diagnosed? *Please* *tick* ***all that apply***

🞏 I attended my GP with urinary symptoms (e.g. urinating frequently, blood in urine).

🞏 I attended my GP with other symptoms (e.g. back pain, joint pain).

🞏 I had no symptoms and my GP offered to test my PSA (blood test) as part of a general health check.

🞏 I had no symptoms and I asked my GP to measure my PSA (blood test).

🞏 I had a PSA test as part of a private health check.

🞏 Other

Please tell us about this:

**8.** Please tell us which treatments you have had following your diagnosis of prostate cancer

*Please tick* ***all*** *the options that apply.*

**A. Have you had surgery (prostatectomy)?** No 🞏 Yes 🞏

If **no**, go to **B**

If **yes**, what type of surgery? Please tick one box

🞏 **Open prostatectomy**

*Operation performed through a cut in the abdomen above the pubic bone area (retropubic prostatectomy) or a cut in the area between the testicles and back passage (perineal prostatectomy).*

🞏 **Laparoscopic (keyhole) prostatectomy**

*Operation performed through small incisions in the abdominal wall.*

🞏 **Robotic prostatectomy**

*Operation performed with the assistance of a surgical robot (Da Vinci prostatectomy).*

🞏 **I don't know what kind of operation I had**

**B. Have you had radiotherapy?** No 🞏 Yes 🞏

If **no**, go to **C**

If **yes**, what type of radiotherapy? Please tick all that apply

🞏 **External beam radiotherapy (with or without hormone treatment)**

*Radiotherapy uses high-energy X-ray beams to treat the whole prostate. This form of treatments includes both 3-dimensional conformal radiotherapy (3D-CRT) and intensity modulated radiotherapy (IMRT).*

🞏 **Permanent seed (low-dose) brachytherapy**

*This involves implanting radioactive seeds into the prostate gland.*

🞏 **Temporary (high-dose) brachytherapy (with or without external beam radiotherapy or hormone treatment)**

*This involves inserting a source of high-dose radiation into the prostate gland for a few minutes.*

🞏 **I don’t know what type of brachytherapy I had**

**C. Have you had any of the below treatments?**

**Please tick all that apply.**

🞏 **High intensity focused ultrasound (HIFU)**

*This treatment uses ultrasound waves to heat and destroy cancer cells in the prostate.*

🞏 **Cryotherapy**

*This treatment uses freezing and thawing to kill the cancer cells in the prostate.*

🞏 **Chemotherapy** (not including hormones)

🞏 **Hormone treatment** (either continuous or on/off treatment)

🞏 **Abiraterone and/or Enzalutamide**

**D. Are doctors and nurses currently monitoring your prostate cancer?**

No 🞏 Yes 🞏

If **no**, go to question **9.a.**

If **yes**, what type of monitoring? Please tick one box.

🞏 **Active Surveillance**

*Surveillance is monitoring of low risk, slow growing* ***localised prostate cancer*** *with the aim of avoiding or delaying* ***curative treatment*** *(e.g. surgery, radiotherapy).This involves having regular tests.*

🞏 **Watchful waiting**

*Watchful waiting is a way of monitoring prostate cancer that isn’t causing any symptoms or problems. The aim is to keep an eye on the cancer over the long term and only having treatment if the cancer deteriorates or the patient gets symptoms. This involves fewer tests than in active surveillance.*

🞏 **Clinical follow-up during or after one of the treatments mentioned above in 2A, B or C**

🞏 **I am unsure about the type of monitoring I am currently having**

Section 3: How things are for you now

We understand that some of the following questions are very sensitive, but we would really appreciate you answering them if possible. As with the rest of the questionnaire, your answers will be kept confidential and no one will be able to identify you.

| 14. Overall, how big a problem has your urinary function been for you during the last 4 weeks?  *Please tick one box.* | |
| --- | --- |
| No problem | 🞏 |
| Very small problem | 🞏 |
| Small problem | 🞏 |
| Moderate problem | 🞏 |
| Big problem | 🞏 |

| 16. Overall, how big a problem have your bowel habits been for you during the last 4 weeks?  *Please tick one box.* | |
| --- | --- |
| No problem | 🞏 |
| Very small problem | 🞏 |
| Small problem | 🞏 |
| Moderate problem | 🞏 |
| Big problem | 🞏 |

Section Seven: Questions about you

| 1. How old are you (in years)? | | |  |  |
| --- | --- | --- | --- | --- |
| 1. What is your legal marital status?   *Please tick* ***one*** *box.* | | | | |
| 🞏 | Married | | | |
| 🞏 | In civil partnership | | | |
| 🞏 | Separated | | | |
| 🞏 | Divorced/dissolved civil partnership | | | |
| 🞏 | Widowed/surviving partner from civil partnership | | | |
| 🞏 | Single (never married/never in civil partnership) | | | |
| 🞏 | Other | | | |
|  | | | | |
|  | | | | |
| 1. What was your employment status at the time of your diagnosis of prostate cancer?   *Please tick* ***one*** *box.* | | | | |
| 🞏 | Full time employment | | | |
| 🞏 | Part time employment | | | |
| 🞏 | Self employed | | | |
| 🞏 | Looking after family/home | | | |
| 🞏 | Retired | | | |
| 🞏 | Unemployed, seeking work | | | |
| 🞏 | Unemployed, unable to work for health reasons | | | |
| 🞏 | Other | | | |
|  | | | | |
|  | | | | |
| 1. What is your employment status currently? *If on sick leave answer in relation to your usual employment status.*   *Please tick* ***one*** *box.* | | | | |
| 🞏 | Full time employment | | | |
| 🞏 | Part time employment | | | |
| 🞏 | Self employed | | | |
| 🞏 | Looking after family/home | | | |
| 🞏 | Retired | | | |
| 🞏 | Unemployed, seeking work | | | |
| 🞏 | Unemployed, unable to work for health reasons | | | |
| 🞏 | Other | | | |
|  | | | | |
|  | | | | |
|  | | | | |
|  | | | | |
|  | | | | |
|  | | | | |
| 1. To which of these ethnic groups would you say you belong?   *Please tick* ***one*** *box.* | | | | |
| White | | | | |
| 🞏 | English/Welsh/Scottish/Northern Irish/British | | | |
| 🞏 | Irish | | | |
| 🞏 | Gypsy or Irish Traveller | | | |
| 🞏 | Any other White background | | | |
| Mixed/Multiple ethnic groups | | | | |
| 🞏 | White and Black Caribbean | | | |
| 🞏 | White and Black African | | | |
| 🞏 | White and Asian | | | |
| 🞏 | Any other Mixed/multiple ethnic background | | | |
| Asian / British Asian | | | | |
| 🞏 | Indian | | | |
| 🞏 | Pakistani | | | |
| 🞏 | Bangladeshi | | | |
| 🞏 | Chinese | | | |
| 🞏 | Any other Asian background | | | |
| Black/African/Caribbean/Black British | | | | |
| 🞏 | Black African | | | |
| 🞏 | Black Caribbean | | | |
| 🞏 | Any other Black / African / Caribbean background | | | |
| Other ethnic group | | | | |
| 🞏 | Arab | | | |
| 🞏 | Any other ethnic group | | | |
|  | | | | |
|  |  | | | |

| 1. Which, if any, of the following conditions do you have?   *Please tick* ***all*** *the boxes that apply.* | | | | | |
| --- | --- | --- | --- | --- | --- |
| 🞏 | A heart condition |  | 🞏 | Kidney disease | |
| 🞏 | Angina |  | 🞏 | Diabetes | |
| 🞏 | High blood pressure |  | 🞏 | Stroke | |
| 🞏 | Asthma or other chronic chest problem |  | 🞏 | Alzheimer’s disease or dementia | |
| 🞏 | Liver disease |  | 🞏 | Epilepsy | |
| 🞏 | Problems with your stomach, bowels or gallbladder |  | 🞏 | Other long standing neurological problem | |
| 🞏 | Problems with your pancreas |  | 🞏 | A diagnosis of arthritis | |

| 1. How tall are you?   feet inches **OR** centimetres 🞏 Don’t know |
| --- |
|  |
| 1. How much do you weigh?   Stone pounds **OR** kilograms grams 🞏 Don’t know |
|  |
| 1. Have you ever in your lifetime seen a health care professional (such as a GP, psychiatrist, psychologist, social worker, counsellor, psychotherapist, mental health nurse, or any other such professional) for problems with your emotions or nerves or your use of alcohol or drugs? |
| 🞏 Yes 🞏 No |
| 1. Do you look after, or give any help or support (not part of your paid employment) to family members, friends, neighbours or others because of either:  - Long term physical or mental health disability, or - Problems relating to old age |
| 🞏 Yes 🞏 No |
